# Matrix Metalloproteinase 13 Is Induced in Fibroblasts in Polyomavirus Middle T Antigen-Driven Mammary Carcinoma without Influencing Tumor Progression

**DOI:** 10.1371/journal.pone.0002959

**Published:** 2008-08-13

**Authors:** Boye S. Nielsen, Mikala Egeblad, Fritz Rank, Hanne A. Askautrud, Caroline J. Pennington, Tanja X. Pedersen, Ib J. Christensen, Dylan R. Edwards, Zena Werb, Leif R. Lund

**Affiliations:** 1 The Finsen Laboratory, Rigshospitalet, Copenhagen, Denmark; 2 Department of Anatomy, University of California San Francisco, San Francisco, California, United States of America; 3 Department of Pathology, Rigshospitalet, Copenhagen, Denmark; 4 School of Biological Sciences, University of East Anglia, Norwich, Norfolk, United Kingdom; 5 Department of Medical Genetics, Ullevål University Hospital and Faculty of Medicine, University of Oslo, Oslo, Norway; Ordway Research Institute, United States of America

## Abstract

Matrix metalloproteinase (MMP) 13 (collagenase 3) is an extracellular matrix remodeling enzyme that is induced in myofibroblasts during the earliest invasive stages of human breast carcinoma, suggesting that it is involved in tumor progression. During progression of mammary carcinomas in the polyoma virus middle T oncogene mouse model (MMTV-PyMT), *Mmp13* mRNA was strongly upregulated concurrently with the transition to invasive and metastatic carcinomas. As in human tumors, *Mmp13* mRNA was found in myofibroblasts of invasive grade II and III carcinomas, but not in benign grade I and II mammary intraepithelial neoplasias. To determine if MMP13 plays a role in tumor progression, we crossed MMTV-PyMT mice with *Mmp13* deficient mice. The absence of MMP13 did not influence tumor growth, vascularization, progression to more advanced tumor stages, or metastasis to the lungs, and the absence of MMP13 was not compensated for by expression of other MMPs or tissue inhibitor of metalloproteinases. However, an increased fraction of thin collagen fibrils was identified in *MMTV-PyMT;Mmp13^−/−^* compared to *MMTV-PyMT;Mmp13^+/+^* tumors, showing that collagen metabolism was altered in the absence of MMP13. We conclude that the expression pattern of *Mmp13* mRNA in myofibroblasts of invasive carcinomas in the MMTV-PyMT breast cancer model recapitulates the expression pattern observed in human breast cancer. Our results suggest that MMP13 is a marker of carcinoma-associated myofibroblasts of invasive carcinoma, even though it does not make a major contribution to tumor progression in the MMTV-PyMT breast cancer model.

## Introduction

The ability of cancer cells to invade or metastasize to distant organs is linked to their ability to traverse the extracellular matrix. The extracellular matrix (ECM) is a heterogeneous protein matrix that consists of a variety of collagens, laminins, fibronectin and proteoglycans [1], [2]. Matrix metalloproteinases (MMP) cleave and remodel ECM components [3], and increased activity of some of the MMPs, including MMP2, 9, and 14 can promote cancer cell invasion *in vivo* [3]–[7].

MMP13 (collagenase-3) was originally identified in human breast cancer tissue [8]. It is secreted from cells as an inactive zymogen that can be activated by the MMP2/MMP14/tissue inhibitor of metalloproteinase (TIMP2) complex [9] or by plasmin [10]. MMP13 acts in the extracellular environment as a potent collagenase capable of degrading a variety of collagens [11]. *Mmp13* mRNA is expressed in a subpopulation of myofibroblasts in invasive ductal breast carcinomas [12], but rarely in normal breast, benign breast lesions and ductal carcinoma in situ (DCIS). Interestingly, the presence of microinvasion in DCIS is associated with focal expression of *Mmp13* mRNA in stromal fibroblasts [12], [13]. Direct comparison of the *Mmp13* mRNA expression pattern with that of the *Mmp2*, *Mmp11* and *Mmp14* mRNAs indicates that *Mmp13* is unique in this respect since these other MMPs are also present in DCIS in the absence of invasion [12]. These observations raise the question as to whether MMP13 is a rate-limiting proteinase that mediates the initial steps in breast cancer invasion.

The role of MMPs and other ECM-degrading proteinases during breast cancer progression has been studied using a variety of murine mammary tumor models [6]. In one model, tumors are induced by expressing the polyoma middle T oncogene under the mouse mammary tumor virus LTR (MMTV-PyMT) [14] leading to early onset hyperplasia in the mammary glands [15]. The PyMT-oncogene induced mammary tumors go through typical pre-invasive, invasive and metastatic phases similarly to human breast cancer [14]. The onset of tumorigenesis in the MMTV-PyMT mouse model is observed at 2–3 weeks after birth, and the non-invasive early stage tumors, mammary intraepithelial neoplasia (MIN), progress into invasive carcinomas at 7–9 weeks after birth [15], [16]. Transition to invasive carcinoma takes place in the central core of the tumors, where epithelial atypia and nuclear pleomorphism first appear [15]. Tumors isolated from 13-week-old mice, late stage tumors, show evidence of dedifferentiation and squamous metaplasia and have spread to the lungs and lymph nodes [14], [15]. The tumors share both morphological and molecular characteristics with human invasive ductal carcinomas [14], [15], [17]–[19]. Furthermore, we have reported that *Mmp2*, *3, 11, 13* and *14* mRNAs are expressed in the stromal compartment in a series of late stage MMTV-PyMT tumors, similar to the expression in human ductal breast cancers [18]. In late stage tumors, *Mmp13* mRNA shows high, focal expression in stromal cell populations [20]. Since this expression pattern is similar to what we have observed in human breast cancers [12], we chose the MMTV-PyMT mouse breast tumor model to test the functional role of MMP13 in breast cancer progression and metastasis.

## Materials and Methods

### Mice

MMTV-PyMT and *Mmp13^−/−^* mice have previously been described [14], [21]. Both strains were backcrossed more than 8 generations to FVB/n. The two strains were intercrossed and the resulting *MMTV-PyMT;Mmp13^+/−^* male offspring were mated with female *Mmp13^+/−^* mice to establish sibling cohorts of *MMTV-PyMT;Mmp13^+/+^* and *MMTV-PyMT;Mmp13^−/−^* females. All animal experiments were conducted according to institutional guidelines and approved by the Danish Animal Experiments Inspectorate. A concurrent health report compliant with the guidelines of the Federation of European Laboratory Animal Science Associations revealed no infections.

### Genotyping

Genotyping was performed on chromosomal DNA purified from tail tips [22] in a single PCR reaction using *MMTV-PyMT* primers, control wt *plasminogen* primers [23] and *Mmp13* primers (MMP13_in5 anti: GGT GGT ATG AAC AAG TTT TCT GAG C, MMP13_in2: CAG ACC CTA CAG TGC CAG ATT TTA G, MMP13_ex5: TGA TGA CGT TCA AGG AAT TCA GTT T). Bands representing *MMTV-PyMT* transgene (159 bp), *plasminogen* (268 bp), wt *Mmp13* (572 bp) and *Mmp13* knock-out allele (672 bp) were identified by agarose gel electrophoresis.

### Quantitative real time PCR

Mice were anesthetized by intraperitoneal administration of a 1∶1 mixture of Dormicum (Roche A/S, Basel, Switzerland) and Hypnorm (Janssen-Cilag Ltd, High Wycombe, UK) and sacrificed by intracardial perfusion with 10 ml ice-cold phosphate-buffered saline (PBS). For analysis of *Mmp13* mRNA expression during normal mammary gland development and MMTV-PyMT tumorigenesis, the #4 mammary glands were isolated from MMTV-PyMT mice and wild type FVB/n mice at 3, 5, 7, 9, 11 and 13 weeks (n = 5 for each age and genotype). For analyses of the expression of other MMPs and TIMPs, tumors from 11-week old *MMTV-PyMT;Mmp13^+/+^* (n = 6) and *MMTV-PyMT;Mmp13^−/−^* mice were used (n = 6). Total mRNA was isolated from 100 mg tissue using the Qiagen RNeasy midi Kit (Qiagen, West Sussex, UK). The purified RNA samples were treated with DNase I for 15 minutes at 37°C to remove residual DNA. Reverse transcription was performed essentially as described [24] followed by TaqMan qPCR performed as described [25] using an ABI7900 (Applied Biosystems, Foster City, CA) and primers specified in [26] except that *Mmp13* primers were chosen from the universal probe library (number 105, Roche Applied Science, Burgess Hill, UK), left: CTT TTC CTC CTG GAC CAA ACT, right: TCA TGG GCA GCA ACA ATA AA. Gene expression levels relative to 18S were calculated.

### In situ hybridization

13-week-old mice were anesthetized as above and perfused intracardially with 10 mL PBS followed by 10 mL of 4% paraformaldehyde (PFA). Mammary tumors were dissected from glands #1–3 and #4, fixed in 4% formalin for 5–7 days at 4°C and paraffin embedded. *In situ* hybridization was performed on samples from *MMTV-PyMT;Mmp13^+/+^* mice (n = 10) with ^35^S labeled RNA probes for MMP13 as described previously [27]. No *Mmp13* mRNA signal was detected in tumors from *MMTV-PyMT;Mmp13^−/−^* mice (n = 4).

### Immunohistochemistry

For immunohistochemical staining of α-smooth muscle-actin (mouse mAb, Dako, Glostrup, Denmark) and CD204 (rat mAb clone 2F8, Serotec, Oxford, UK), sections were heat-treated at 98°C in TEG buffer (10 mmol/L Tris, 0.5 mmol/L EGTA, pH 9.0) for 5 minutes in a T/T Micromed microwave processor (Milestone, Sorisol, Italy). The α-smooth muscle-actin antibody was mixed with biotinylated anti-mouse Fab fragments using the Animal Research Kit (Dako) and used for staining following the recommendations of the manufacturer. The rat mAb against CD204 was detected with rabbit anti-rat antibodies followed by Envision-Rabbit reagent (Dako). All antibody incubation steps were performed in an OptiMax automated immunostainer (BioGenex, San Ramon, CA). Immunohistochemical staining combined with *in situ* hybridization was performed on mouse glands #1–3 tumor samples from *MMTV-PyMT;Mmp13^+/+^* mice (n = 6) as described previously [12]. The sections were developed with DAB chromogene, fixed for 40 minutes in 4% PFA, and washed three times with sterile water. After dehydration, the sections were incubated with ^35^S-labeled *Mmp13* antisense probes and further treated according to the *in situ* hybridization as described previously [27].

### Morphometric analysis of mammary gland whole mounts

The #4 mammary glands were isolated from 4- and 6-week-old *MMTV-PyMT;Mmp13^+/+^* mice (n = 4 and n = 4, respectively) and *MMTV-PyMT;Mmp13^−/−^* mice (n = 3 and n = 7, respectively) and prepared for whole mount as described [28]. The whole mounts were photographed using a DFC320 CCD camera (Leica Microsystems, Wetzlar, Germany) and the ductal epithelial invasion determined by measuring the distance from the branch point closest to the nipple to the three most far-reaching ducts and the edge of the fat pad. The penetration is presented as the mean of the three measures of ductal penetration related to the length to the edge of the fat pad. All measurements were done using the Leica IM500 software.

### Tumor growth and lung metastasis

Tumor growth in *MMTV-PyMT;Mmp13^+/+^* mice (n = 38) and *MMTV-PyMT;Mmp13^−/−^* mice (n = 25) was followed by weekly palpations of all 10 mammary glands. The length and width of all palpable tumors were measured by caliper. The tumor volume (assuming that tumors took the shape of an ellipsoid) was calculated using the formula: *V* = (*π*/6)×*W* ^2^×*L*, where *L* = length and *W* = width. At 13 weeks, *MMTV-PyMT;Mmp13^+/+^* mice (n = 35) and *MMTV-PyMT;Mmp13^−/−^* mice (n = 25) were anesthetized and perfusion fixed (three mice had been terminated before due to extensive tumor burden). The lungs were removed for determination of the metastasis burden by stereological analysis using Cavalieri's principle as described previously [29].

### Tumor grading

Nuclear grading of the MMTV-PyMT tumors was performed on haematoxylin and eosin (H&E) stained sections from the #1–3 tumors of *MMTV-PyMT;Mmp13^+/+^* mice (n = 34) and *MMTV-PyMT;Mmp13^−/−^* mice (n = 24). We defined grade I tumors as tumors with adenoma and atypia in Mammary Intraepithelial Neoplasia (MIN)-like foci and containing up to 5% of MIN with grade II nuclear pleiomorphy. Grade II tumors (“Early carcinoma” [15]) were tumors with widespread invasive carcinoma with grade II nuclear pleiomorphy and contained areas with MIN Grade I/II. Grade III tumors (“Late carcinoma”) were tumors with focal or widespread invasive carcinoma with grade III nuclear pleiomorphy that also contained areas with Grade I and II carcinoma. Most tumors with grade III carcinoma showed areas of squamous metaplasia.

### Quantification of angiogenesis

Mice were anesthetized as above and 100 µL of 1 mg/mL FITC-conjugated *Lycopersicon esculentum* (tomato) lectin (Vector labs) injected *i.v.* in the tail vein. After 5 min, the mice were cardiac perfused with 4% PFA in PBS at 120–140 mm Hg until the flow-through fluid was clear. Tumors were dissected and post-fixed in 4% PFA overnight, incubated in increasing sucrose concentrations (12%, 15%, 18% in PBS), embedded in Tissue-Tek O.C.T. compound and thick sections (30–40 µm) cut. The tissue sections were stained with 1∶500 solution of Propidium Iodide in PBS (Molecular probes, P-3566) for 1 hour and the slides were mounted with Gel/Mount (Biomeda, #M01). Confocal image stacks with 1-µm spacing were collected from the most vascularized area in the middle of the tumors using a 20x lens with a Solamere micro-lensed spinning disk confocal microscope (Solamere Technologies, Salt Lake City UT) equipped with an intensified charge-coupled device (ICCD) camera (XRMega-10EX S-30, Stanford Photonics, Palo Alto, CA). The acquired images were analyzed with Bitplane Imaris version 5.5 for Windows software using ‘filament tracer’ with an approximate filament diameter set at 1.5. Minor manual corrections of the computerized filaments were uniformly done. Vessel (segment) average diameter, length and volume, were exported to a Microsoft Excel file and analyzed using GraphPad 4 statistical software. Eight tumors from *MMTV-PyMT;Mmp13^+/+^* and nine tumor from *MMTV-PyMT;Mmp13^−/−^* were analyzed. All image acquisition and analysis was done blindly.

### Cell proliferation analysis

*MMTV-PyMT;Mmp13^+/+^* mice (n = 19) and *MMTV-PyMT;Mmp13^−/−^* mice (n = 19) were injected *i.p.* with 2 mg bromodeoxyuridine (BrdU, Sigma-Aldrich) 2 hours before euthanasia. The mice were anesthetized, perfusion fixed, the tumors isolated and fixed in 4% PFA and finally paraffin embedded as described above. For immunohistochemistry, we used a rat mAb against BrdU at 2 µg/mL (clone BU1/75-ICR1, Abcam, Cambridge, UK). The BrdU labeled cells were counted as a percentage of the total number of epithelial tumor cells using CastGRID (Visiopharm, Hørsholm, Denmark) essentially as previously described [30]. In brief, frames of 9391 µm^2^ were systematically but randomly placed over the BrdU stained sections with a final magnification of 658 on the monitor. All BrdU positive neoplastic cells within the frames were counted to give total numbers varying from 250–400. The total number of neoplastic cells within the frames was estimated based on the usage of systematic and randomly positioned reference points [30].

### Collagen evaluation

Serial sections (3-µm thick) from mammary tumor glands #1–3 obtained from 13-week-old *MMTV-PyMT;Mmp13^+/+^* mice (n = 26) and *MMTV-PyMT;Mmp13^−/−^* mice (n = 21) were stained with haematoxylin and eosin (H&E) or picrosirius red (PSR) [31]. Areas with invasive tumor were identified in H&E stained sections. The same areas were then analyzed in the PSR stained sections by obtaining 2.3×1.8 mm images with a charge-coupled device (CCD) camera (Leica) under linearly polarized light using a Leica DMRBE microscope. In linearly polarized light, thick collagen bundles appear orange-red and thin collagen fibers green [32]. Images were analyzed with MetaMorph software for collagen content. Total collagen was identified with Hue-Saturation-Intensity (HSI) intervals 0–256, 1–256 and 70–256, respectively. For thin fibrillar collagen, HSI settings of 26–83, 70–135, and 57–155 were applied to all the images. All image acquisition and analysis was done blindly. The area covered by total collagen, thin fibrillar collagen, and the fraction of thin fibrillar collagen was calculated and used for statistical analysis (t-test).

### Statistics

For statistical analyses of the tumor burdens obtained by caliber measurements, the total tumor volumes were logarithmically transformed and analyzed using a general linear model with repeated measures. Only measurements obtained from week 7 to 13 were included in this analysis. Estimates were obtained using generalized estimating equations (SAS v9.1). Statistical interaction between genotype and time were evaluated for the entire observation period considering repeated measures (SAS). For statistical analyses of the metastasis burdens, the values were logarithmically transformed and analyzed using a general linear model.

## Results

### Mmp13 is expressed by myofibroblasts in invasive stage MMTV-PyMT tumors

**Figure 1:**
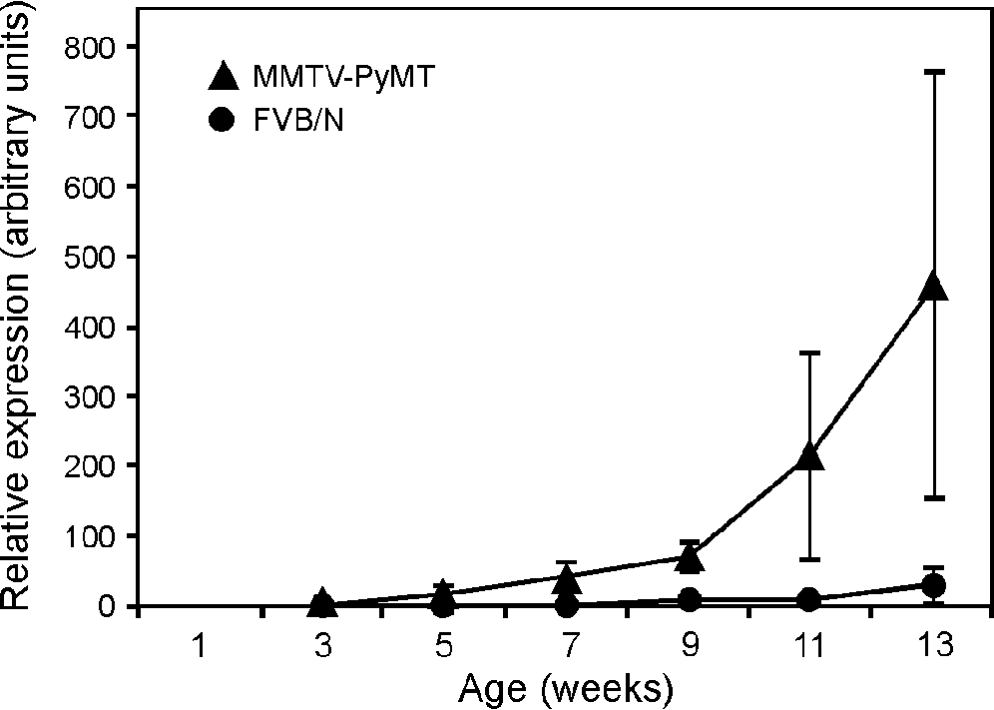
Expression of Mmp13 mRNA is induced at 7 weeks of age in MMTV-PyMT tumors. *Mmp13* mRNA levels determined by qPCR during normal development (•) and MMTV-PyMT induced tumor progression (▴) in the #4 mammary gland (n = 6±1 for each time point). Values represent mean±standard error of the mean (SEM).

*Mmp13* mRNA is expressed by a subpopulation of myofibroblasts in human invasive ductal breast carcinomas, but is rarely expressed in normal breast, benign breast lesions and ductal carcinoma in situ (DCIS) [12]. To determine if the expression level of MMP13 changes during tumor progression in the MMTV-PyMT mouse model, *Mmp13* mRNA levels were assessed in tumor tissue from mice at 3-13 weeks of age by qPCR. The level of *Mmp13* mRNA was highly increased in MMTV-PyMT tumors after 7-weeks of age, with the highest levels found in older mice, corresponding to late stage tumors (Fig. 1). *Mmp13* mRNA levels were low in normal mammary glands at all ages. The increase in *Mmp13* mRNA thus took place during the time of transition to invasive stages in this tumor model [15].

**Figure 2:**
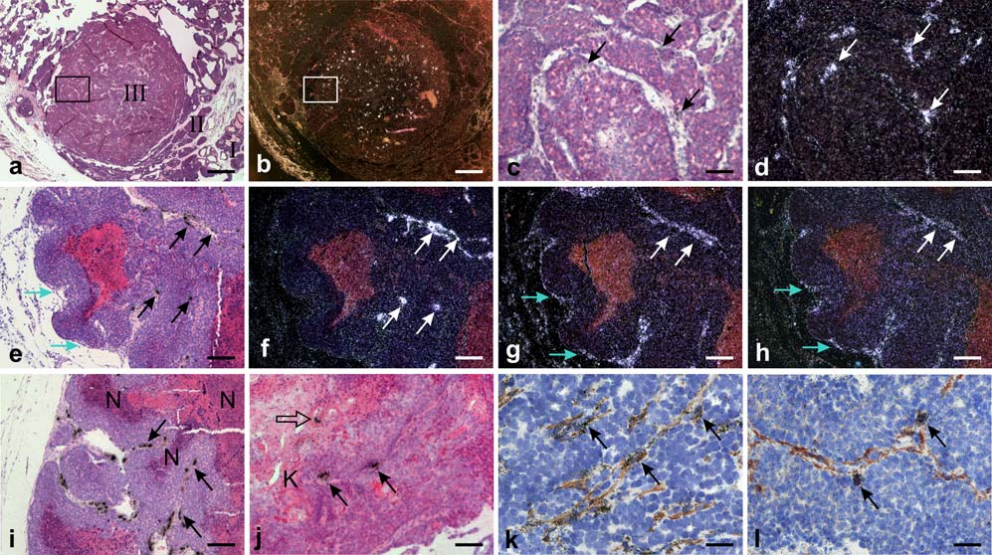
Mmp13 mRNA is expressed by a subset of α-smooth muscle actin positive carcinoma-associated fibroblasts. MMTV-PyMT tumors from 13-week-old mice were processed for *in situ* hybridization for *Mmp13* (a–e, i–l), *Mmp2* (g), and *Mmp14* (h). For clarity the hybridization signals (silver grains) are shown with dark-field illumination in b, d, f, g, and h. (c) and (d) are higher magnification of the area with grade III invasive carcinoma framed in (a) and (b). *Mmp13* mRNA signal is seen in the areas with grade III invasive carcinoma (c–d, arrows), but not in the surrounding areas with grade I and II MIN (indicated by I and II in a and b). *Mmp13* mRNA expression is restricted to the tumor core (f, white arrows), whereas Mmp2 (g) and Mmp14 (h) mRNAs are seen also in the tumor periphery (blue arrows, f, g, and h are adjacent sections). Stromal septae with *Mmp13* mRNA positive signal (i, arrows) are seen in areas close to necrotic tissue (N). *Mmp13* mRNA signal in tumor areas with squamous metaplasia showing keratinized (K) epithelium (j, arrows). Expression of *Mmp13* mRNA is seen in α-smooth muscle-actin immunoreactive myofibroblasts (k and l, arrows), in some areas restricted to myofibroblasts in the delicate strands of stromal tissue within the cellular tumor (l, arrows). All sections were counter-stained with haematoxylin and eosin except (k and l) that were stained with haematoxylin only. Bars: a, b: 700 µm; c, d: 100 µm; e–i: 400 µm; j: 200 µm; k, l: 50 µm.

To determine where *Mmp13* was expressed, we performed *in situ* hybridization in tumors from 13-week-old mice, containing different stages of nuclear grading. The *Mmp13* mRNA signal was seen in stromal fibroblast-like cells located in the tumor core in areas with invasive grade II and III carcinoma (“late carcinoma”), but was generally absent in the tumor periphery that was dominated by normal-looking mammary tissue, hyperplasia or MIN grade I and II (Fig. 2a–d). In particular, *Mmp13* mRNA positive cells were often seen in stromal branches located close to the tumor cores with focal necrosis (Fig. 2i). Sporadic *Mmp13* mRNA positive single cells and small cell clusters were also found in areas with strongly keratinized, squamous metaplastic changes (Fig. 2j). This focal expression of *Mmp13* in the tumor core was very different from the expression of *Mmp2* and *Mmp14*, which were found throughout the tumor stroma, including at the tumor periphery (Fig. 2 e–h), and in areas with hyperplasia, grade I and grade II MIN (not shown).

The localization of the cells that expressed *Mmp13* suggested that they were myofibroblasts. Using combined *in situ* hybridization and immunohistochemical staining, we verified that most of the *Mmp13* mRNA positive cells were positive for the myofibroblast marker α-smooth muscle-actin (Fig. 2k), paralleling the expression we found in human breast cancers [12]. Myofibroblasts are prevalent in the invasive MMTV-PyMT tumors, but only a small subpopulation was positive for *Mmp13* mRNA. We noted that some of the *Mmp13* mRNA positive myofibroblasts were located in narrow bands between tumor cell clusters (Fig. 2l). CD204 positive macrophages did not express *Mmp13* mRNA and the location of the *Mmp13* mRNA positive cells was not consistent with myoepithelial cells (data not shown), supporting our conclusion that the stromal *Mmp13* mRNA expressing cells were myofibroblasts.

### Early tumor formation does not require MMP13 in MMTV-PyMT

**Figure 3:**
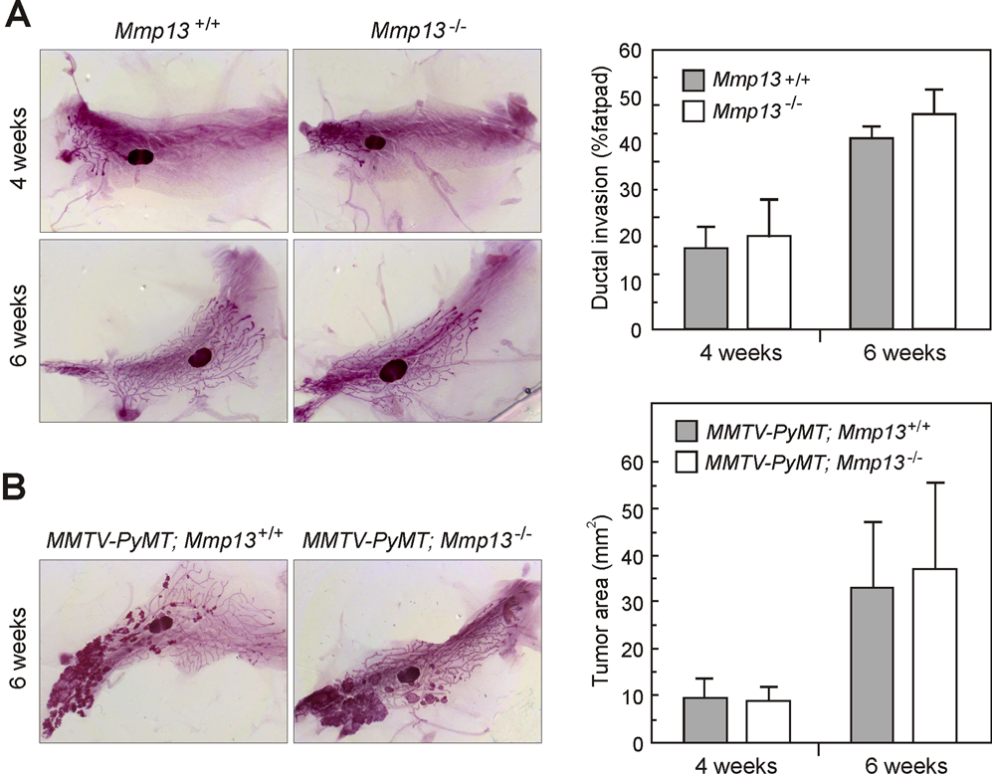
Mammary gland development and early tumor development is not influenced by MMP13. (A) Morphometric analysis of ductal epithelial penetration during normal mammary gland development. Representative whole mount-stained #4 mammary glands from *Mmp13^+/+^* (n = 4 and n = 4 at 4 and 6 weeks of age, respectively), and *Mmp13^−/−^* mice (n = 3 and n = 7 at 4 and 6 weeks of age, respectively. The distances from the nipple to the three most far-reaching ducts and to the edge of the fat pad were measured. The mean ductal epithelial penetration as percentage of the whole fat pad is shown with the SEM. (B) Morphometric analysis of early MMTV-PyMT tumor growth. Representative whole mount-stained #4 gland tumors from *MMTV-PyMT;Mmp13^+/+^* and *MMTV-PyMT;Mmp13^−/−^* mice at 6-weeks-of-age. The tumor area was measured in 4- and 6-week-old *MMTV-PyMT;Mmp13^+/+^* (n = 5 and n = 7, respectively) and *MMTV-PyMT;Mmp13^−/−^* mice (n = 3 and n = 10, respectively) and are presented as mean with the SEM.

The expression levels and patterns of *Mmp13* mRNA suggested that the proteinase would play a role in the transition to the invasive stages. To determine the role of MMP13 in mouse mammary tumor progression, we took a genetic approach using *Mmp13^−/−^* mice. We verified that the absence of MMP13 did not affect the development of the mammary epithelium (Fig. 3A) and therefore would not affect tumor progression indirectly by influencing the number of mammary epithelial cells. We next crossed the MMTV-PyMT mice with *Mmp13^−/−^* mice and measured the total hyperplastic area on whole-mounts of mammary glands from mice at age 4 or 6 weeks, before the transition to invasive carcinoma. No difference was found between *MMTV-PyMT;Mmp13^+/+^* and *MMTV-PyMT;Mmp13^−/−^* mice (Fig. 3B), consistent with the lack of *Mmp13* mRNA expression at the early cancer stage.

### MMP13 is not required for tumor growth, tumor vascularization or metastasis in MMTV-PyMT mammary carcinomas

**Figure 4:**
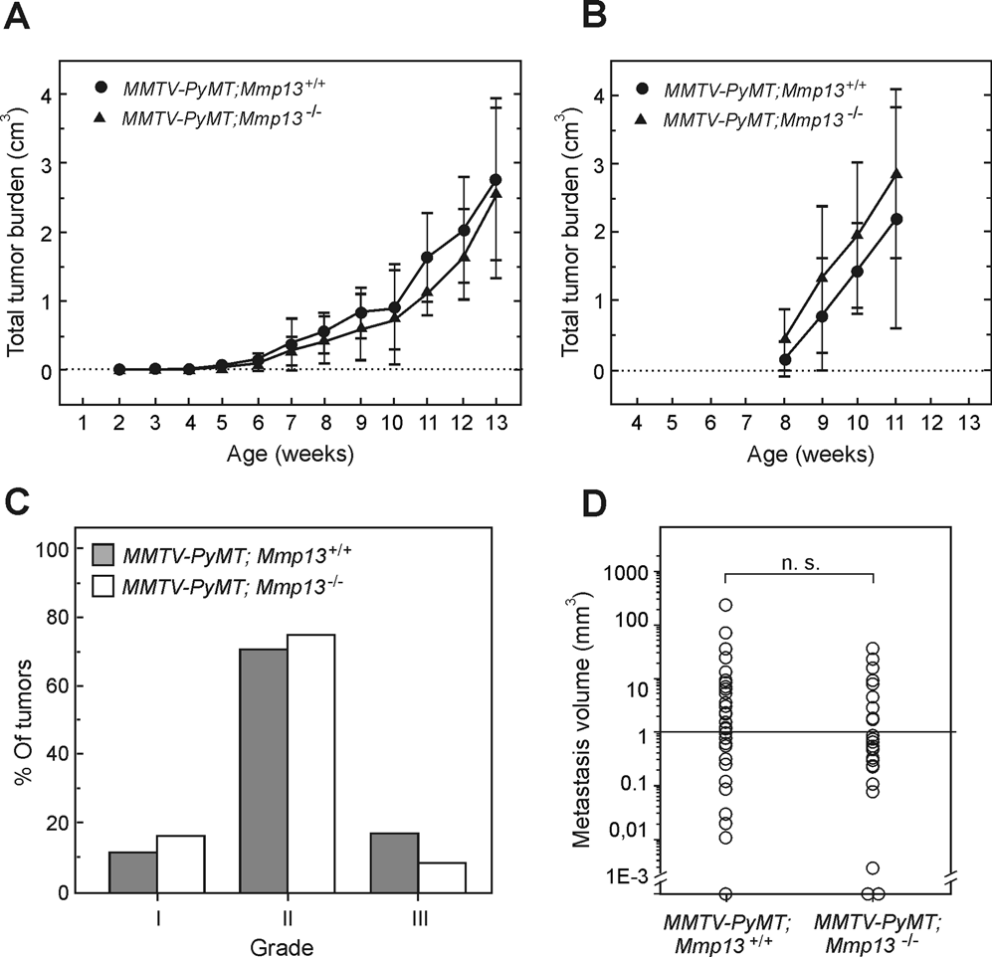
MMTV-PyMT primary tumor growth and metastasis is not influenced by MMP13. (A) MMTV-PyMT tumor growth determined by weekly caliber measurements. Total tumor volumes are presented as mean +/− the SEM. (•) *MMTV-PyMT;Mmp13^+/+^*, n = 38, (▴) *MMTV-PyMT;Mmp13^−/−^*, n = 25 . Three *MMTV-PyMT;Mmp13^+/+^* mice were terminated before the end of the experiment due to extensive tumor burden, one at 8 weeks and two after 12 weeks. (B) MMTV-PyMT tumor growth determined by weekly caliber measurements in another cohort. (•) *MMTV-PyMT;Mmp13^+/+^*, n = 6, (▴) *MMTV-PyMT;Mmp13^−/−^*, n = 7. The total weight of the tumors at 11 weeks was 1.42g±0.22 SEM for the *MMTV-PyMT;Mmp13^+/+^* mice and 1.44g±0.16 SEM for the *MMTV-PyMT;Mmp13^−/−^* mice. (C) Nuclear grading of the MMTV-PyMT tumors. Nuclear grading was performed on representative H&E stained sections from mammary tumors glands #1–3 from 13-week-old *MMTV-PyMT;Mmp13^+/+^* (n = 34) and *MMTV-PyMT;Mmp13^−/−^* (n = 24) mice by a pathologist (FR) unaware of the genotypes. Nuclear grading was independent of genotype. (D) Lung metastasis volumes determined by stereology using Cavalieri's principle. The total metastasis volume was determined in lungs obtained from the 13-week-old mice by an observer unaware of the genotypes. Median values are 1.2 mm^3^ in the *MMTV-PyMT;Mmp13^+/+^* (n = 35) and 0.7 mm^3^ (n = 25) in *MMTV-PyMT;Mmp13^−/−^* mice, respectively.

**Figure 5:**
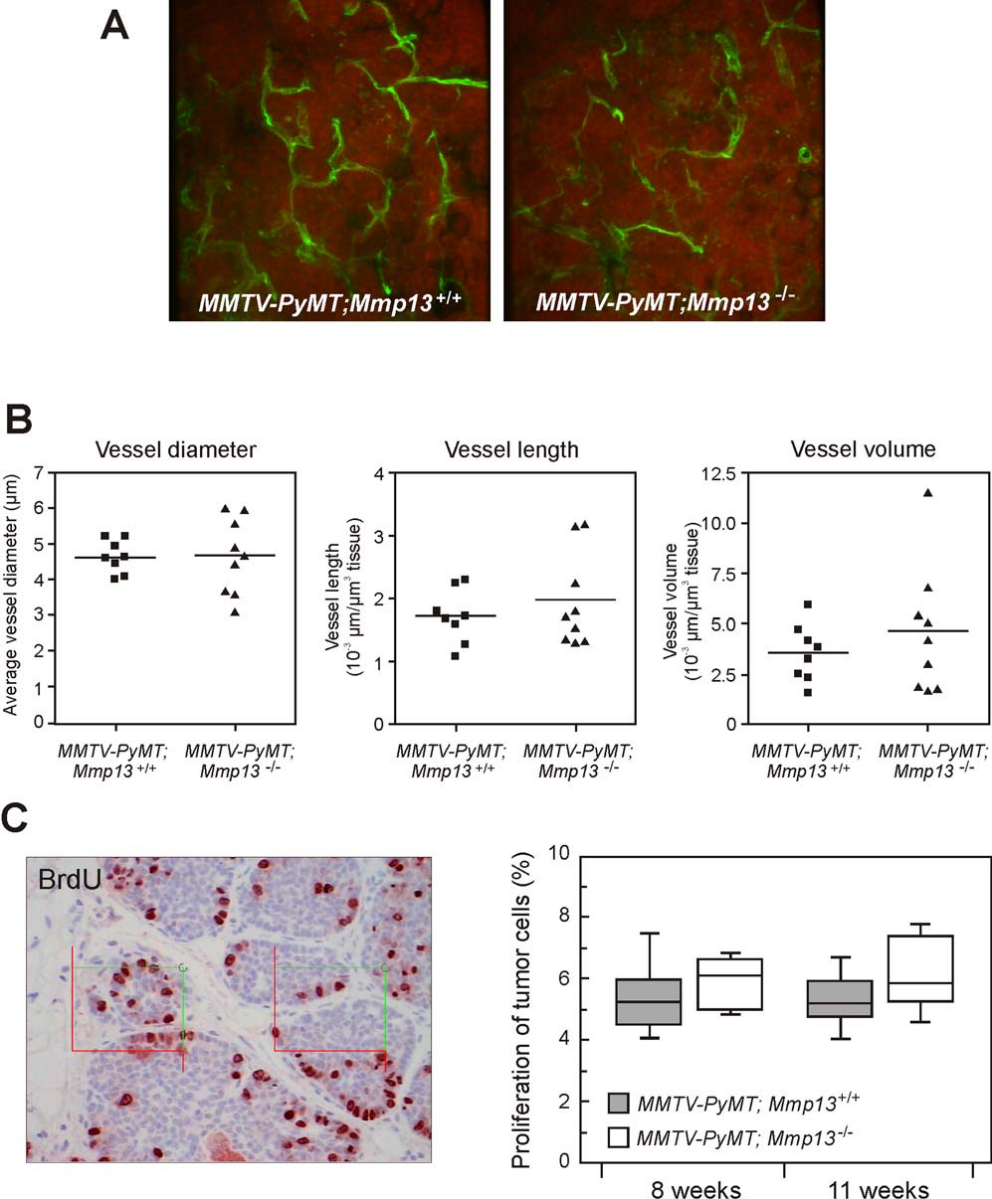
Vascularization of MMTV-PyMT tumors is not affected by MMP13. (A) Maximum intensity projections of confocal image stacks from tumor sections showing cell nuclei (red, propidium iodide stained) and vasculature (green, with FITC-conjugated lycopersicon esculentum lectin). (B) No significant differences were observed in vessel diameter (p = 0.95), length (p = 0.4) or volume (p = 0.4) when comparing vessels in tumors from *MMTV-PyMT;Mmp13^+/+^* (n = 9) and *MMTV-PyMT;Mmp13^−/−^* mice (n = 9, t-tests). All analyses were done blindly. (C) Morphometric analysis of the proliferation rate in the MMTV-PyMT tumors. Representative immunohistochemical staining for BrdU in an MMTV-PyMT tumor with counting frames applied to the tissue sections (left). Tumor cell proliferation was determined by an observer unaware of the genotypes in one #1–3 gland and one #4 gland tumor from each mouse. *MMTV-PyMT;Mmp13^+/+^*, n = 8 and n = 11 at 8 and 11 weeks, respectively, *MMTV-PyMT;Mmp13^−/−^*, n = 7 and n = 12 at 8 and 11 weeks, respectively (three samples were excluded due to poor morphology). The proliferation rates are shown as the percentage of the total number of neoplastic cells with medians 50% (box) and 95% inclusion intervals (right).

*Mmp13* mRNA was induced in tumors from mice of 7–9 weeks-of-age, and we therefore expected that MMP13 would be required in the later stages of tumorigenesis, possibly affecting tumor growth and metastasis. We followed tumor growth in a cohort of littermate MMTV-*PyMT;Mmp13^+/+^* (n = 38) and *MMTV-PyMT;Mmp13^−/−^* females (n = 25) by weekly palpations and caliper measurements until 13 weeks of age, when tumors were isolated for histological examination and the lungs isolated to determine metastasis burden. A minor reduction in the growth of the tumors in *MMTV-PyMT;Mmp13^−/−^* mice were seen at 8, 9, 11, 12 weeks (Fig. 4A), but this was not reproduced in a different cohort (Fig. 4B). Furthermore, there were no differences in cancer cell proliferation between tumors from *MMTV-PyMT;Mmp13^+/+^* and *MMTV-PyMT;Mmp13^−/−^* mice at 8 or 11 weeks of age as determined by BrdU-labeling (Fig. 5C). In addition, histological examination and nuclear grading of the tumors revealed no significant differences in the percentages of tumors that had progressed to grade III, late-stage carcinoma (Fig. 4C, 8% of the *MMTV-PyMT;Mmp13^−/−^* vs. 18% of MMTV-*PyMT;Mmp13^+/+^* tumors). Furthermore, analyses of H&E stained sections revealed no differences in the presence of necrosis or stroma between tumors from *MMTV-PyMT;Mmp13^−/−^* and MMTV-*PyMT;Mmp13^+/+^* mice (not shown). Finally, no effects were seen on tumor vascularization, when analyzing vessel diameter, length, or volume in 3D reconstructions of vessels in tumors of lectin-perfused mice (Fig. 5A, B). Thus, the absence of MMP13 did not influence progression in the primary tumors.

Since MMP13 is upregulated concomitantly with the onset of cancer cell dissemination in the MMTV-PyMT model [16], we determined the metastatic burden in the lungs from end-stage *MMTV-PyMT;Mmp13^+/+^ and MMTV-PyMT;Mmp13^−/−^* mice using a morphometric approach [29]. Although there was a trend for a reduced lung metastatic volume in the absence of MMP13, this was not significant (Fig. 4D, 1.2 mm^3^ for *MMTV-PyMT;Mmp13^+/+^*, n = 35; 0.7 mm^3^ for *MMTV-PyMT;Mmp13^−/−^*, n = 25, *p* = 0.34). We therefore conclude that the absence of MMP13 does not affect lung metastasis in the MMTV-PyMT breast cancer model.

### There are no compensatory changes in expression of MMPs or TIMPs in MMTV-PyMT;Mmp13^−/−^ tumors

**Figure 6:**
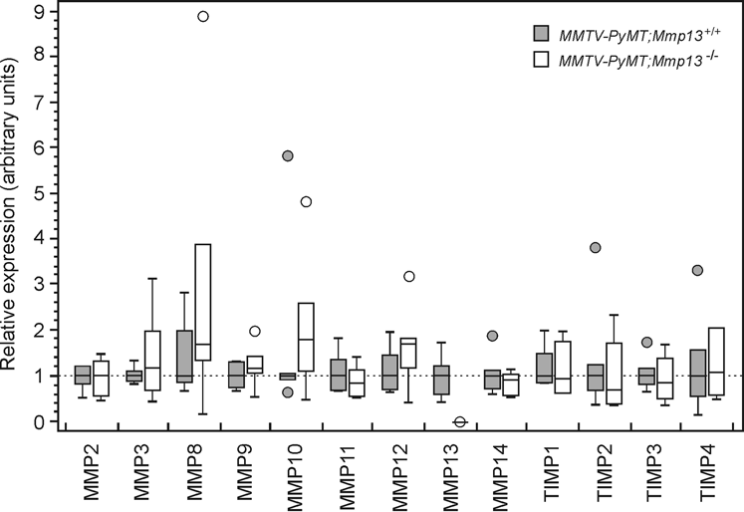
Absence of MMP13 is not compensated for by changes in mRNA expression of other MMPs or TIMPs. Expression of *Mmp* and *Timp* mRNAs, relative to 18S ribosomal RNA, in *MMTV-PyMT;Mmp13^+/+^* (white boxes, *n* = 6) and *MMTV-PyMT;Mmp13^−/−^* (gray boxes, *n* = 6) tumors. In the box and whiskers plot, all values are normalized to the median value of the *MMTV-PyMT;Mmp13^+/+^* group. Horizontal lines indicate the median and the boxes include the 25^th^ and 75^th^ percentiles. The lines outside the boxes correspond to highest or lowest values observed when these did not fall within the quartiles, and circles (white or gray) correspond to outlier values.

Since we found no changes in tumor progression in the absence of MMP13, we explored the possibility that the lack of MMP13 activity was compensated for by changes in expression of other MMPs or in their inhibitors, the TIMPs. However, there were no significant differences in the mRNA levels for MMP2, -3, -8, -9, -10, -11, -12, -13, -14 or TIMP1, -2, -3, or -4 in tumors with and without MMP13 from 11-week-old mice by qPCR (Fig. 6).

### Increased amounts of thin collagen fibers is found in the absence of MMP13 in invasive areas of MMTV-PyMT tumors

**Figure 7:**
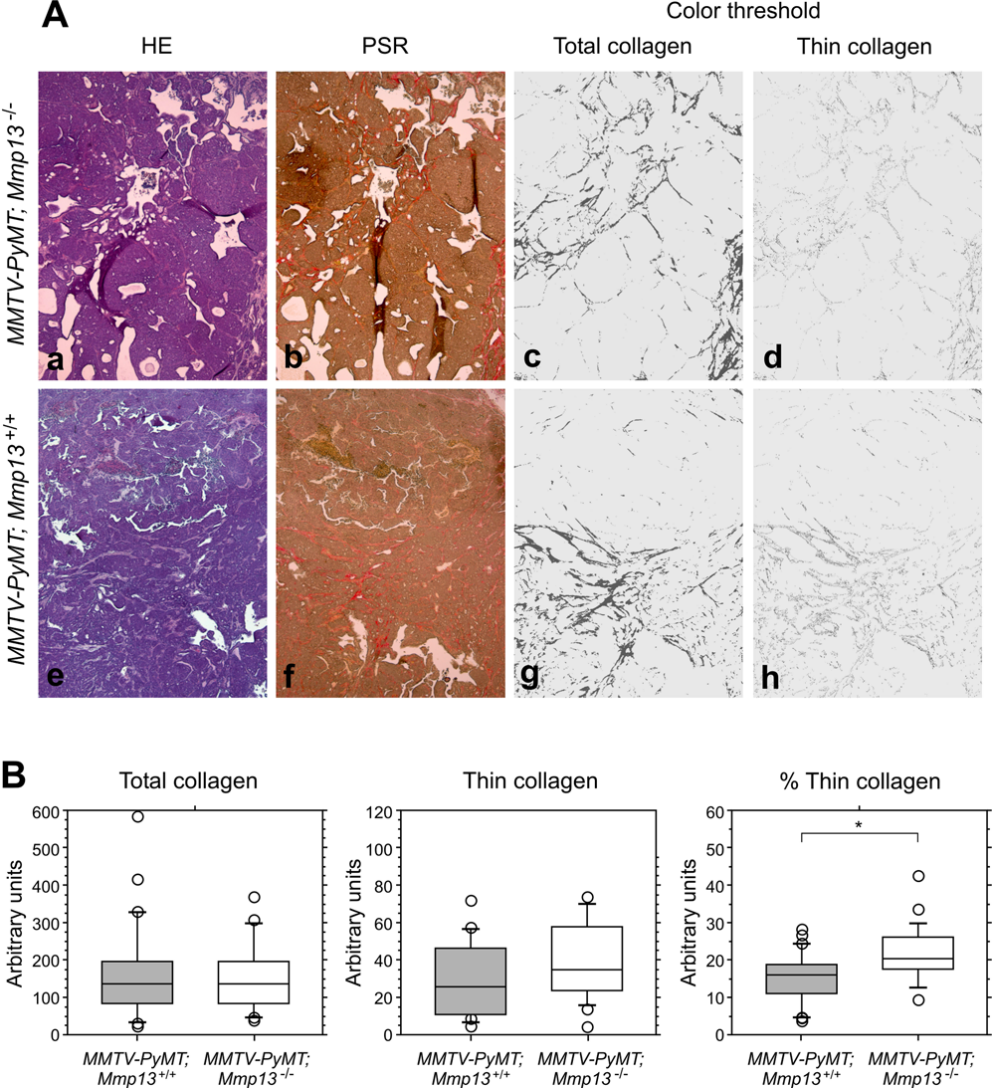
Absence of MMP13 influences fibrillar collagen in the MMTV-PyMT tumor stroma. (A) Tissue sections from *MMTV-PyMT;Mmp13^+/+^* (a-d) and *MMTV-PyMT;Mmp13^−/−^* (e–h) tumors stained with haematoxylin and eosin (a,e) or picrosirius red (b,f), the latter illuminated with linearly polarized light. Color threshold images of total collagen (c,g) and thin collagen fibers (d,h) of the images in b and f, respectively. (B) Box plots of total collagen (left), thin collagen fibers (middle) and the ratio between thin collagen fibers and total collagen (right, *p<0.01, t-test) measured in *MMTV-PyMT;Mmp13^+/+^* (gray boxes, *n* = 26) and *MMTV-PyMT;Mmp13^−/−^* (white boxes, *n* = 21) tumors as shown in the A panel.

To determine whether any effects of MMP13 could be seen on its major substrate, fibrillar collagen, we evaluated the fibrillar collagen content in invasive areas of the tumors by picrosirius red staining of tumor sections (Fig. 7A). Although total collagen was not affected by the absence of MMP13, the fraction of thin collagen fibers relative to total collagen was increased significantly by ∼30% in the invasive areas of tumors from MMTV-*PyMT;Mmp13^−/−^* mice (Fig. 7B, t-test, p = 0.007). Thus, MMP13 played a role in collagen metabolism in invasive areas of MMTV-PyMT tumors, but this did not affect tumor progression.

## Discussion

In this study, we analyzed the effects of absence of MMP13 activity on the progression of MMTV-PyMT mammary carcinoma. Our previous studies of human DCIS with early invasion led us to hypothesize that MMP13 would be a fibroblast-derived, potential rate-limiting proteinase in the transition from non-invasive to invasive breast carcinoma [12]. Interestingly, a recent study reported upregulation of several MMPs, including MMP13, at the transition from non-invasive to invasive breast cancer cells in an *in vitro* model [33]. However, although *Mmp13* mRNA also was strongly induced in a subpopulation of myofibroblasts in MMTV-PyMT mammary carcinomas, concurrent with early transition to invasive carcinoma, there were no effects on tumor cell proliferation, tumor growth, lung metastasis, vascularization or differentiation of the primary tumors by the absence of MMP13. We excluded that the lack of effects was due to compensatory transcriptional changes of other MMPs or their inhibitors expressed in the MMTV-PyMT tumors.

MMP13 has a broad substrate profile that includes several fibrillar collagens, tenascin, fibronectin and proteoglycans [11], [34], [35]. *Mmp13* mRNA was found in carcinoma-associated fibroblasts located in the central areas of the MMTV-PyMT tumors, where tumor dedifferentiation and invasion is prominent. In these areas, we found increased thin collagen fibers relative to total collagen in *MMTV-PyMT;Mmp13^−/−^* compared to *MMTV-PyMT;Mmp13^+/+^* tumors, indicating that MMP13 influences collagen fiber formation and/or metabolism of fibrillar collagen in the MMTV-PyMT stroma. Mice lacking *Mmp13* display no major phenotypic abnormalities [21], [36], but a significant contribution of MMP13 in collagen metabolism has been reported during bone development [21], [36], skeletal regeneration [37] and in atherosclerotic plaques [38].

We found that most of the *Mmp13* mRNA expressing cells in the MMTV-PyMT tumors are carcinoma-associated myofibroblasts. The myofibroblast is an abundant cell type in the MMTV-PyMT tumors (this study) as it is in invasive human breast cancers [39]. There are several reports suggesting that carcinoma-associated myofibroblasts promote cancer progression [40]–[42]. In addition, proteins expressed by myofibroblasts, such as PAI-1 and uPA, are associated with poor prognosis for breast cancer patients [43], [44]. It is unknown what induces *Mmp13* in the breast carcinoma-associated myofibroblasts, but the restricted expression of *Mmp13* in dense fibrous stromal septae within the central parts of the invasive MMTV-PyMT tumors suggests a locally expressed tumor-cell derived factor. *In vitro* studies have shown that IL-1α, IL-1β, and transforming growth factor-β1, in particular, can induce *Mmp13* mRNA expression in fibroblasts [45]. Interestingly, TGFβ signaling through fibroblast-expressed TGFβ receptor type II has a considerable impact on tumor growth [46]. *Mmp13* mRNA expressing myofibroblasts are often found in the tumor core close to areas with necrosis suggesting that MMP13 may be upregulated in response to hypoxia for stimulation of angiogenesis. Indeed, *Mmp13* mRNA is induced by hypoxia in various cell lines [47]–[49] including fibroblasts [49], and vasculature of ossification sites and migration of endothelial cells into cartilage is impaired in *Mmp13^−/−^* mice [50]. However, our measurements of vascular diameter, length and volumes in MMTV-PyMT tumors did not reveal any effect of MMP13 on tumor vascularization.

It has been reported that *Mmp8* and *Mmp9* mRNAs are upregulated during bone development in the ossification sites in *Mmp13^−/−^* mice [36] and that *Mmp8* mRNA is upregulated during skin wound healing in *Mmp13^−/−^* mice [51]. We tested whether the mRNA expression levels of several MMPs were changed in *MMTV-PyMT;Mmp13^−/−^* tumors, but found no significant changes. We found that tumor progression in the MMTV-PyMT model was associated with strong upregulation of the *Mmp13* mRNA level as compared to the normal mammary gland. Preliminary results suggest that *Mmp10* and *Mmp12* mRNA also are upregulated, while *Mmp2*, -3, -8, -9, -11, and -14 are not (TXP, CP, DE, unpublished data). This suggests that the three MMPs are upregulated by a common mechanism and potentially implicates MMP10 and MMP12 in tumorigenesis in the MMTV-PyMT model even though *MMP10* and *MMP12* mRNAs were not significantly upregulated in the *MMTV-PyMT;Mmp13^−/−^* tumors compared to MMTV-PyMT wild type tumors. However, we cannot exclude that differences exist at the level of enzyme activity or that other classes of proteolytic enzymes compensate for the absence of MMP13. The expression of *Mmp13* mRNA is strongly restricted compared to the expression of *Mmp2* and *Mmp14* mRNAs that are expressed in a broader group of (myo)fibroblasts. We have previously reported that mRNAs for several MMPs, including *Mmp2*, *Mmp3*, *Mmp11* and *Mmp14* are expressed in the MMTV-PyMT tumor stroma [18]. Of these MMPs, MMP2 and MMP14 may have substrates in common with MMP13, for example, both are also collagenases *in vivo* [3]. Therefore MMP2, MMP14 or the activity of other MMPs may compensate for the lack of MMP13 in the *MMTV-PyMT;Mmp13^−/−^* tumors.

It is possible that MMP13 plays both promoting and inhibiting roles during cancer progression, resulting in a neutral net effect in its absence in the MMTV-PyMT model. Tumor promoting and inhibiting functions have been found for MMP9 in the human papilloma virus (HPV) 16 skin cancer model where the absence of *Mmp9* reduced the number of tumors, but the tumors that formed were more aggressive [4]. A tumor protective role of a number of matrix degrading proteases, including MMP8 and MMP12, has been described [52]. These studies emphasize the need for better understanding the role not only of the individual proteases but also of their separate activities in cancer progression.

Breast cancer progression in humans may have a larger contribution from the stromal environment than murine tumors, since human tissue have a more fibroblast-rich stroma than murine [6]. Nevertheless, the MMTV-PyMT breast cancer model shares many histological and molecular characteristics with human luminal breast cancer [14], [15], [17], but it differs from human ductal carcinoma in one interesting aspect: the myoepithelial cells, which represent a cellular barrier for tumor cells and are an important source for basement membrane, are partly lost early during *MMTV-PyMT* oncogene-induced tumor progression [15]. The absence of an intact myoepithelial barrier may lead to pre-invasive precursor lesions different from human DCIS and render MMP13 a dispensable proteinase in the critical transition phase from non-invasive to invasive carcinoma. Thus, despite the presence of a heterogeneous stromal environment and the appearance of a considerable myofibroblast population in the MMTV-PyMT tumors, myofibroblasts may play a minor role for the overall progression of the MMTV-PyMT tumor compared to human breast cancer. Therefore, models in which transition from DCIS to invasive carcinoma occurs similar to in human tumors are needed to definitely rule out a role of MMP13 in breast cancer.

In conclusion, we found that the absence of MMP13 in the MMTV-PyMT mice did not result in any differences in tumor progression to invasive and metastatic breast carcinoma. Carcinoma-associated fibroblasts have been shown to facilitate tumor progression through stimulation of angiogenesis, growth factor secretion and invasion [53]–[55]. However, there are no good markers allowing systematic sub-classification of these cells. Thus, even though MMP13 itself apparently is dispensable for tumor progression in the MMTV-PyMT model, its restricted expression in myofibroblasts in invasive regions suggests that it marks a subpopulation of carcinoma-associated fibroblasts that are involved in the transition to invasive carcinoma.
